# Memorial to Congress

**Published:** 1847-12

**Authors:** 


### MEMORIAL TO CONGRESS.

The great importance to the profession of Medicine, and to the afflicted who are subjects of treatment, of the matter contained in the following memorial to Congress, will, we hope, induce physicians generally to copy, procure signatures to, and forward it to their respective Representatives and Senators in Congress.

The proceedings of the New York College of Pharmacy in our last number, under the head of "Adulterations of Medicine," sets forth more fully the important necessity of some action on this behalf.

It is respectfully suggested that the Medical Societies in their organized capacities, might do much towards forwarding the objects of the memorial, which we take from the *N. Y. Journal of Medicine.*

[MEMORIAL.]

*To the Honorable the Senate and House of Representatives:*

The Memorial of the College of Pharmacy, of the City of New York, respectfully represents:

That large quantities of sophisticated and misnamed Chemicals and Pharmaceutical preparations are daily imported, not only to the injury of the Custom House revenue, and of the honest importer, but of dangerous effect upon the health and lives of all who require the aid of medicines such as they purport to be, throughout the country.

That with some unprincipled foreign manufacturers, aided and abetted by dealers of a kindred stamp in this country, it is a regular and systematic business to make different qualities of various medical preparations for the American market—the better kinds for the Atlantic cities, and others very much inferior "for the West," meaning thereby, our Western States. The latter are generally altogether unlike what they purport to be, are quoted at about half prices, and are unfit for any use whatever.

That of an almost indefinite number of spurious and misnamed articles, may be specified Iodide of Potassium, for which the Bromide is substituted, in whole or in part, and other salts, of an entirely different character, are also mixed in large proportions.

Blue Pill comes to us containing from 10 down to 7½ per cent. of Mercury, (the officinal proportion being 33⅓ per cent.) mixed with blue clay and Prussian blue, to give it density and color—the following being the composition, by the analysis of our Professor Reid, of a lot which passed the Custom House about the first of August.

| | |
|---|---|
| Mercury, - - - - | 7·5 |
| Earthy clay, - - - | 27·0 |
| Prussian blue, used in coloring, - | 1·5 |
| Sand in combination with the clay, - | 2·0 |
| Soluble saccharine matters, - | 34·0 |
| Insoluble organic matters, - - | 12·0 |
| Water, - - - - | 16·0 |
| | 100·0 |

Very large quantities of Rhubarb, much decayed, the better parts of which are dark colored, with scarcely any taste or smell, having probably been exhausted to make extract, come from England, invoiced there from 1½ to 3 pence sterling per lb.—entirely worthless trash. It is intended and used for powdering, color being given to it by tumeric, etc.

The article called Oxyd of Zinc on the English labels, is generally *Carbonate* of Zinc, being imported at a price which precludes the possibility of honest preparation.

All that is received under the name of Milk of Sulphur, except small lots expressly ordered from some manufacturing establishment of honorable character, contains from 80 to 95 per cent of Sulphate of Lime, (Plaster of Paris.)

Many of the foreign Extracts are not what they purport to be, and are entirely worthless as medicines.

Opium is often invoiced at one third the value of good quality, and is found upon examination not to be worth even that. The same may be said of Scammony.

The Essential Oils generally, the Salts of Quinine, Morphine, and all the more costly chemicals, are greatly adulterated.

These are some of the most important that come to us from abroad. Upon them the Physician depends, in many cases, for his success in controlling disease, we need hardly say, with frequent disappointment, the sufferings of the sick being protracted, other complications of disorder allowed to supervene, and life itself often sacrificed.

That numerous instances frauds, of the kind here spoken of, have been publicly exposed in the newspapers, by the authority of this College. Such exposures have undoubtedly proved of excellent service, so far as related to each particular fraud published and the extent reached by the publication, but this unpleasant and burthensome duty is greatly inadequate to the correction of the evil.

Your memorialists therefore pray your Honorable Bodies that a law may be enacted, declaring that all imported articles intended for medical use, which may appear to the proper Custom House Officer to be spurious, counterfeited, or adulterated, shall be subject to competent inspection, and if found to be of base character, confiscated and destroyed.

And your memorialists, &c.

